# Supplementary figures and images for: Exosome-delivered circRPS5 inhibits the progression of melanoma via regulating the miR-151a/NPTX1 axis
Source: PLoS One. 2023 Jun 29;18(6):e0287347. doi: 10.1371/journal.pone.0287347 (PMC10310028; doi:10.1371/journal.pone.0287347)

**Fig. 1B/1D**

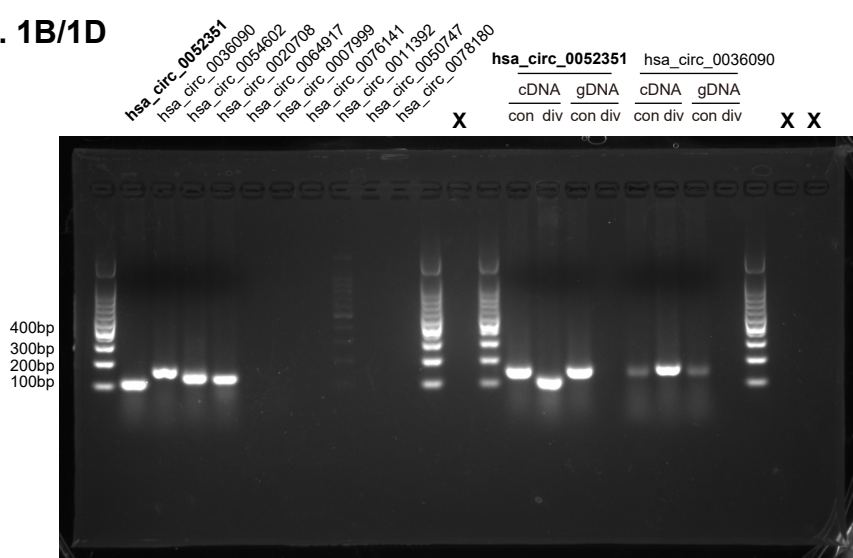

**Fig. 3C**

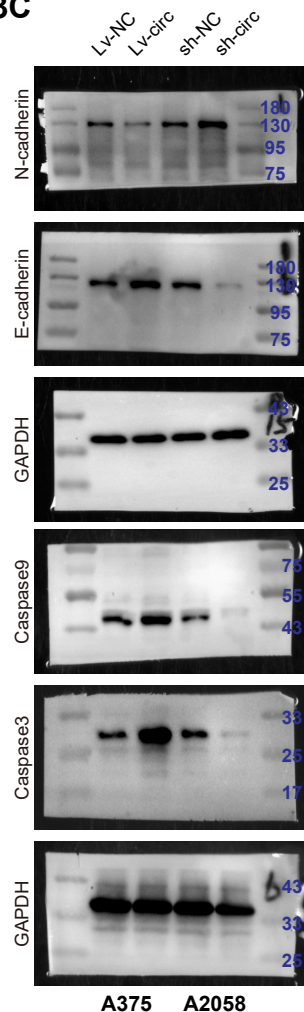

**Fig. 4C**

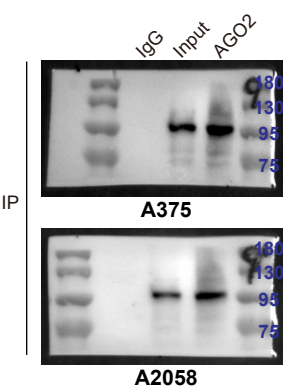

**Fig. 5F**

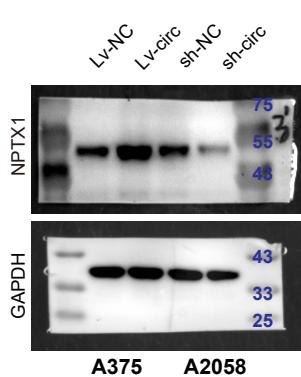

**Fig. 5J**

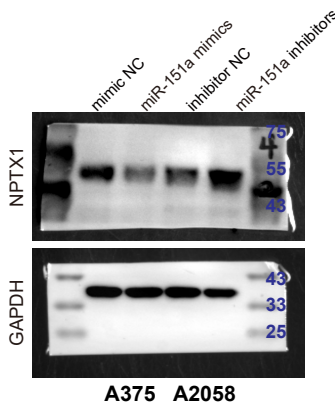

**Fig. 5K**

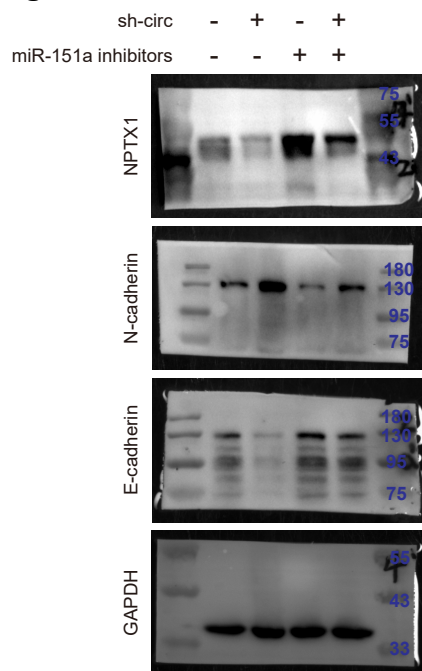

**Fig. 6B**

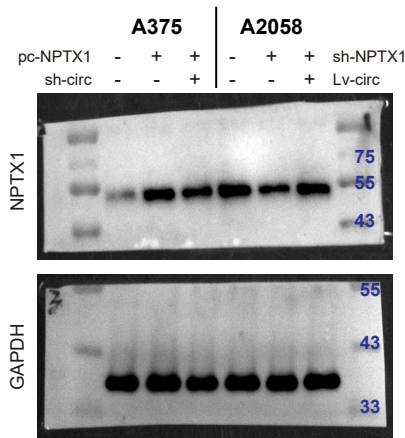

**Fig. 7C**

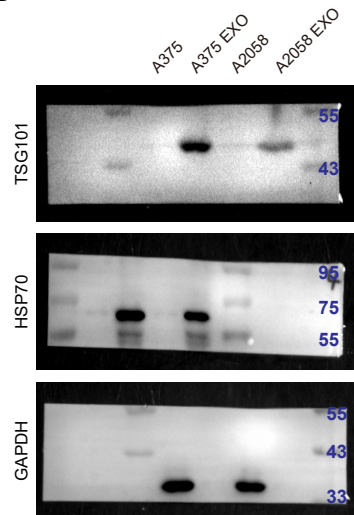

Supplement: S1 Raw images — (PDF) [file pone.0287347.s001.pdf]
