# Supplementary material for: Exosome-delivered circRPS5 inhibits the progression of melanoma via regulating the miR-151a/NPTX1 axis
Source: PLoS One. 2023 Jun 29;18(6):e0287347. doi: 10.1371/journal.pone.0287347 (PMC10310028; doi:10.1371/journal.pone.0287347)
Supplement: Attachment — Submitted filename: Minor Revision.docx [file pone.0287347.s002.docx]

In this paper entitled “Exosome-delivered circRPS5 inhibits the progression of melanoma via regulating the miR-151a/NPTX1 axis” the authors have investigated the role of a novel exosome-delivered circular RNA named circRPS5 that plays a regulatory role in melanoma progression as well as proliferation, migration and invasion through the miR151a/NPTX1 pathway.

The study is well performed and the results are very interesting to be potentially useful for clinical application suggesting that circRPS5 could be a novel therapeutic target for melanoma therapy.

However the authors should respond to minor points. Particularly:

1) the authors through qRT-PCR demonstrate that circRPS5 is downregulated in two melanoma cell lines, A375 and A2058. In order to investigate the biological functions of circRPS5 in melanoma, the authors apply the circRPS5 overexpressing system in A375 cell lines and the circRPS5 knockdown system in A2058 cell line. Why the authors decide to proceed differently in the two cell lines of melanoma although in Fig.1E they show that the level of downregulation of the circRPS5 is equal in the two cell lines?

2) In the section Materials and Methods, in particular in the paragraph referring to the western blotting method, the authors write: "Equal amounts of protein were separated by SDS-PAGE gel...". Authors should specify the amount of protein loaded.

In conclusion the manuscript could be accepted for publication on PLOS ONE after minor revision.
